# Screening of Natural Organic Volatiles from *Prunus mahaleb* L. Honey: Coumarin and Vomifoliol as Nonspecific Biomarkers

**DOI:** 10.3390/molecules16032507

**Published:** 2011-03-16

**Authors:** Igor Jerković, Zvonimir Marijanović, Mladenka Malenica Staver

**Affiliations:** 1 Faculty of Chemistry and Technology, University of Split, N. Tesle 10/V, 21000 Split, Croatia; 2 Marko Marulić Polytechnic in Knin, P. Krešimira IV 30, 22300 Knin, Croatia; 3 Department of Biotechnology, University of Rijeka, S. Krautzeka bb, 51000 Rijeka, Croatia

**Keywords:** *Prunus mahaleb* L. honey, headspace-solid-phase microextraction (HS-SPME), ultrasonic solvent extraction (USE), gas chromatography and mass spectrometry (GC, GC-MS), coumarin, vomifoliol

## Abstract

Headspace solid-phase microextraction (HS-SPME; PDMS/DVB fibre) and ultrasonic solvent extraction (USE; solvent A: pentane and diethyl ether (1:2 v/v), solvent B: dichloromethane) followed by gas chromatography and mass spectrometry (GC, GC-MS) were used for the analysis of *Prunus mahaleb* L. honey samples. Screening was focused toward chemical composition of natural organic volatiles to determine if it is useful as a method of determining honey-sourcing. A total of 34 compounds were identified in the headspace and 49 in the extracts that included terpenes, norisoprenoids and benzene derivatives, followed by minor percentages of aliphatic compounds and furan derivatives. High vomifoliol percentages (10.7%–24.2%) in both extracts (dominant in solvent B) and coumarin (0.3%–2.4%) from the extracts (more abundant in solvent A) and headspace (0.9%–1.8%) were considered characteristic for *P. mahaleb* honey and highlighted as potential nonspecific biomarkers of the honey’s botanical origin. In addition, comparison with *P. mahaleb* flowers, leaves, bark and wood volatiles from our previous research revealed common compounds among norisoprenoids and benzene derivatives.

## 1. Introduction

The diversity of *Prunus* spp. L. nectariferous honey plants from the family *Rosaceae* in Croatia is pronounced, and includes the following species: *P. armeniaca* L., *P. avium* L., *P. cerasifera* Ehrh., *P. cerasus* L., *P. dulcis* (Mill.) D. A. Webb, *P. fruticosa* Pall., *P. mahaleb* L., *P. padus* L., *P. spinosa* L. and *P. tenella* Batsch. [1]. Mahaleb (*Prunus mahaleb* L.) kernels [2] contain coumarin, herniarin (7-methoxycoumarin), dihydrocoumarin and a small amount of amygdaline (mandelonitrile-β-gentio-bioside). Furthermore, the kernels contain ca. 40% fatty oil with unusual conjugated fatty acids, such as 9,11,13-octadecatrienoic acid (*cis*-, *trans*-, *trans*- and *cis*-, *trans*-, *cis*-isomers) and *ca*. 31% proteins [2]. The ratio of coumarin and herniarin in different parts of this plant and their biosynthesis pathways were investigated previously [3]. The plant is robust and insensitive to diseases and is used as a stock in the grafting of cherry and marasca. [4]. The kernel (embryo) of the seed is soft-textured and tastes bitter and aromatic, especially after chewing for some time, when a subtle bitter almond flavour develops. Mahaleb kernels are used in folk medicine as a tonic and antidiabetic, as well as a flavouring agent. [5]. The volatile compounds of *P. mahaleb* leaves, flowers, stem-bark and wood were isolated by steam distillation and subsequently analysed by GC and GC-MS [6]. One hundred and thirty components were identified. Aliphatic hydrocarbons (e.g. C_20_, C_21_ and C_28_), alcohols, carbonyls, fatty acids (e.g. dodecanoic, tretradecanoic, hexadecanoic and linoleic acids), terpenes, C_13_-norisoprenoids and phenylpropane derivatives (e.g. coumarin) were detected. *P. mahaleb* is also listed as a botanical source of honey in Europe [7], but with no detailed data on the chemical characterization of the honey is available. Wild-growing *P. mahaleb* in Croatia served as the main nectar source of unifloral honey samples for the presented research. 

In addition to traditional approaches, examination of the volatiles profile might be considered as a strategy enabling honey authentication, since its composition is known to vary widely with the floral origin and way of processing [8]. Finding specific or nonspecific biomarker compounds is a powerful tool in the determination of the honey botanical origin [8,9,10,11]. For this purpose, the volatile fraction of *P. mahaleb* honey was investigated in present paper for the first time by means of headspace solid-phase microextraction (HS-SPME) and ultrasonic solvent extraction (USE) followed by gas chromatography and mass spectrometry analyses (GC, GC-MS). The comparison with results of our previous research [6] on volatiles from *P. mahaleb* flowers, leaves, bark and wood is also presented.

## 2. Results and Discussion

Evaluation of the sensory characteristics as well as the melissopalynological analyses confirmed the floral origin of all *Prunus mahaleb* L. honey samples. In particular, from the melissopalynological analyses 24 pollen types were identified in the sediment of the honey samples, with the presence of *Prunus mahaleb* as the main plant species (up to 29%) followed by *Erica arborea* L. (up to 13%), *Viburnum tinus* L. (up to 11%), *Robinia pseudoacacia* L. (up to 9%), *Acer* spp. L. (up to 7%), *Olea europaea* L. (up to 5%). Minor pollen percentages from *Salix* spp. L., *Pistacia* spp., *Fraxinus* spp. L., *Asparagus acutifolius* L., *Prunus* spp. L., *Centaurea* spp. L., *Laurus nobilis* L., *Liliaceae*, *Pinus* spp. *Salvia officinalis* L., *Asteraceae*, *Aesculus hippocastanum* L., *Asphodelus* spp. L., *Brassicaceae*, *Quercus ilex* L., *Cistus* spp. L., *Hippocrepis* spp. L. and others were found in the samples. All the samples were analyzed by HS-SPME and USE followed by GC and GC-MS.

### 2.1. Organic Volatiles Isolated by HS-SPME

In the recent past, instrumental analysis of the honeys has become dominated by headspace analysis, particularly the rapid, solvent-less, and easy-to-use HS-SPME extraction technique [12,13]. Several fiber coatings with different polarity and extraction mechanism have been used for the extraction of honey headspace compounds [14,15]. In this research, polydimethylsiloxane/divinylbenzene (PDMS/DVB) fibre was selected.

Terpenes were the most abundant headspace organic volatiles in the samples (Table 1): linalool (4.0%–6.6%), three isomers of lilac aldehyde (1.0%–11.7%), three isomers of lilac alcohol (0.1%–1.2%), *cis*- and *trans*-linalool oxide (0.6%–3.1%). Linalool, *cis*- and *trans*-linalool oxides are common terpenes of honey volatiles [15]. The second group of abundant volatiles were norisoprenoids: C_9_-norisoprenoids [such as 4-ketoisophorone (5.0%–7.4%), α-isophorone (2.1%–6.5%), 2-hydroxyisophorone (0.0%–0.9%) or 2-hydroxy-4-ketoisophorone (0.0%–0.6%)] and C_10_-norisoprenoid safranal (0.0%–1.0%). 

**Table 1:** *Prunus mahaleb* L. honey volatiles composition obtained by HS-SPME followed by GC and GC-MS analysis.

No.	Compound	RI	Min.	Max.	Av.	SD.
1	Dimethyl sulfide	<900	0.0	0.9	0.43	0.45
2	2-Methylbutanal	<900	0.0	0.9	0.37	0.47
3	2,5-Dimethylfuran	<900	0.0	2.9	1.30	1.47
4	3-Methylpentan-3-one	<900	2.0	2.4	2.20	0.20
5	Octane	<900	0.0	1.7	0.83	0.85
6	Furfural	<900	0.0	0.4	0.23	0.21
7	3-Methylpentan-1-ol	<900	0.0	0.4	0.20	0.20
8	3-Methylpentanoic acid	941	1.0	1.3	1.13	0.15
9	Benzaldehyde	965	2.0	4.5	3.37	1.27
10	Phenylacetaldehyde	1048	0.5	2.8	1.43	1.21
11	*trans*-Linalool oxide (furan type)	1076	2.0	3.1	2.53	0.55
12	*cis*-Linalool oxide (furan type)	1091	0.6	1.0	0.80	0.20
13	Linalool	1101	4.0	6.6	5.13	1.33
14	Nonanal	1102	0.0	0.8	0.37	0.40
15	Hotrienol	1106	0.5	1.9	1.10	0.72
16	α-Isophorone	1124	2.1	6.5	4.10	2.23
17	Lilac aldehyde (isomer I)	1151	1.0	4.9	3.30	2.04
18	4-Ketoisophorone	1152	5.0	7.4	6.17	1.20
19	Lilac aldehyde (isomer II)	1159	6.1	11.7	9.00	2.81
20	Lilac aldehyde (isomer III)	1174	2.2	4.9	3.37	1.39
21	3,5,5-Trimethylcyclohexan-1,4-dione	1177	0.0	1.8	0.93	0.90
22	Benzoic acid	1181	0.2	1.4	0.87	0.61
23	Safranal	1206	0.0	1.0	0.40	0.53
24	Lilac alcohol (isomer I)	1210	0.1	1.1	0.47	0.55
25	Lilac alcohol (isomer II)	1220	0.0	0.6	0.27	0.31
26	Lilac alcohol (isomer III)	1222	0.1	0.6	0.30	0.26
27	Car-2-en-4-one^*^	1228	0.0	1.2	0.67	0.61
28	2-Hydroxy-3,5,5-trimethyl-cyclohex-2-en-1,4-dione(2-Hydroxy-4-ketoisophorone)	1245	0.0	0.6	0.30	0.30
29	5-Hydroxymethylfurfural	1254	0.7	1.8	1.17	0.57
30	4-Methoxybenzaldehyde (4-Anisaldehyde)	1264	1.4	10.9	4.93	5.20
31	1-Methoxy-4-propylbenzene	1307	0.5	5.3	2.60	2.46
32	3,4,5-Trimethylphenol^**^	1336	1.1	3.6	2.40	1.25
33	Coumarin	1444	0.9	1.8	1.23	0.49
34	Hexadecanoic acid	1981	0.5	3.0	1.50	1.32

RI = retention indices on HP-5MS column; A = solvent-free HS-SPME; ^*^ - tentatively identified; ^**^ - correct isomer not identified.

Identified headspace phenylpropane derivatives were: coumarin (0.9%–1.8%), benzaldehyde (2.0%–4.5%), phenylacetaldehyde (0.5%–2.8%), benzoic acid (0.2%–1.4%), 4-methoxybenzaldehyde (1.4%–10.9%) and 3,4,5-trimethylphenol (1.1%–3.6%). Benzaldehyde, phenylacetaldehyde, 4-methoxybenzaldehyde, 3,4,5-trimethylphenol and coumarin were found among *P. mahaleb* volatiles [6] isolated by hydrodistillation.

### 2.2. Organic Volatiles Isolated by USE

During the course of our well established methodology of ultrasonic assisted honey extraction using different solvents [16] we performed USE separately applying two solvents: solvent A – mixture of pentane and diethyl ether (1:2 v/v) and solvent B – dichloromethane. Identified compounds in both extracts are listed in Table 2. The C13-norisoprenoid vomifoliol was the most abundant single compound, with ranges of 10.7%–13.8% (solvent A) and 21.6%–24.2% (solvent B). It was not found in the headspace, probably due to its polarity and less volatility, as we already reported in our previous screening on *Mentha* spp. L. honey volatiles [17]. In distinction from the headspace composition, only 4-ketoisophorone (0.2%–1.9%; 0.1%–0.6%) and 4-hydroxy-α-isophorone (0.3%–0.6%; 0.0%–0.1%) were found among C9-norisoprenoids. Methyl syringate was the second abundant compound (4.5%–6.8%; 3.8%–4.2%). Aromatic acids and esters (Table 2) were the most widespread in solvent A and solvent B extracts respectively: benzoic acid (2.5%–4.9%; 0.3%–0.8%), phenylacetic acid (0.8%–1.4%; 0.1%–0.2%), vanillin (0.0%–0.5%; 0.0%–0.2%), 4-methoxybenzoic acid (0.4%–4.7%; 0.2%–1.8%), methyl 4-hydroxybenzoate (0.0%–0.5%; 0.0%–0.2%), methyl vanillin (0.0%–0.9%; -), methyl 4-hydroxy-3-methoxybenzoate (0.9%–2.9%; 0.1%–0.5%), methyl 4-methoxy-benzoate (2.0%–3.3%; 0.3%–1.0%), methyl 2,5-dihydroxybenzoate (0.0%–1.0%; 0.0%–0.3%), 4-hydroxybenzoic acid (0.4%–3.7%; -), 3,4-dimethoxybenzoic acid (-; 0.3%–1.0%). Other present phenyl-propane aldehydes and alcohols were: benzaldehyde (0.0%–0.3%; 0.0%–0.2%), benzyl alcohol (0.0%–0.2%; 0.0%–0.1%), phenylacetaldehyde (0.0%–0.4%; 0.0%–0.1%), 4-methoxybenzaldehyde (1.0%–1.9%; 0.1%–0.3%), 4-methoxybenzyl alcohol (0.0%–0.3%; -), 4-methoxy-2-phenylethanol (0.3%–0.5%; 0.0%–0.1%), 4-hydroxybenzaldehyde (0.0%–0.3%; 0.0%–0.1%). Coumarin was found with higher percentages in solvent A (1.1%–2.4%) in comparison with solvent B (0.3%–0.4%).

**Table 2:** *Prunus mahaleb* L. honey volatile organic composition obtained by USE with two solvents followed by GC and GC-MS analysis.

No.	Compound	RI		Area percentages (%)								
				Solvent A					Solvent B			
				Min.	Max.	Av.	SD.		Min.	Max.	Av.	SD.
1	3-Methylbutanoic acid	< 900		-	-	-	-		0.0	0.1	0.07	0.06
2	1,3-Dimethylbenzene^**^	< 900		-	-	-	-		0.0	0.1	0.07	0.06
3	Nonane	900		0.1	0.8	0.33	0.32		-	-	-	-
4	3-Methylpentanoic acid	941		0.0	1.1	0.35	0.51		0.0	0.2	0.10	0.10
5	Benzaldehyde	965		0.0	0.3	0.15	0.13		0.0	0.2	0.13	0.12
6	Benzyl alcohol	1037		0.0	0.2	0.08	0.10		0.0	0.1	0.07	0.06
7	Pantoic lactone	1044		-	-	-	-		0.0	0.1	0.07	0.06
8	Phenylacetaldehyde	1048		0.0	0.4	0.18	0.17		0.0	0.1	0.05	0.06
9	4,5-Dimethyl-2-formylfuran	1078		0.1	0.5	0.25	0.19		0.1	0.6	0.30	0.22
10	Methyl 2-furoate	1084		0.1	0.3	0.18	0.10		0.2	0.7	0.45	0.24
11	4-Ketoisophorone	1152		0.2	1.9	0.98	0.85		0.1	0.6	0.40	0.25
12	Benzoic acid	1181		2.5	4.9	3.65	1.10		0.3	0.8	0.58	0.21
13	(*E*)-3,7-dimethylocta-1,5-diene-3,7-diol(Terpenediol I)	1191		0.1	0.2	0.13	0.05		0.0	0.2	0.10	0.10
14	2,3-dihydrobenzofuran (coumaran)	1245		0.0	0.7	0.25	0.33		-	-	-	-
15	5-Hydroxymethylfurfural	1251		1.0	2.6	2.13	0.75		1.0	5.0	3.33	1.70
16	4-Methoxybenzaldehyde (4-Anisaldehyde)	1262		1.0	1.9	1.53	0.38		0.1	0.3	0.23	0.10
17	Phenylacetic acid	1283		0.8	1.4	1.10	0.26		0.1	0.2	0.15	0.06
18	4-Methoxybenzyl alcohol	1292		0.0	0.3	0.15	0.13		-	-	-	-
19	1-Methoxy-4-propylbenzene	1307		0.0	1.6	0.88	0.80		0.0	0.4	0.20	0.16
20	4-Hydroxy-3,5,5-trimethyl-cyclohexan-1-one-2-ene (4-Hydroxy-α-isophorone)	1320		0.3	0.6	0.45	0.13		0.0	0.1	0.07	0.06
21	3,4,5-Trimethylphenol^**^	1333		0.5	0.8	0.65	0.13		0.5	1.5	0.80	0.48
22	4-Methoxy-2-phenylethanol	1377		0.3	0.5	0.43	0.10		0.0	0.1	0.05	0.06
23	4-Hydroxybenzaldehyde	1393		0.0	0.3	0.13	0.25		0.0	0.1	0.05	0.06
24	Vanillin	1412		0.0	0.5	0.25	0.24		0.0	0.2	0.10	0.08
25	Coumarin	1444		1.1	2.4	1.83	0.56		0.3	0.4	0.35	0.06
26	4-Methoxybenzoic acid	1452		0.4	4.7	3.35	1.99		0.2	1.8	1.03	0.66
27	Methyl 4-hydroxybenzoate	1482		0.0	0.5	0.23	0.22		0.0	0.2	0.10	0.10
28	3,4-Dimethoxybenzaldehyde (methyl vanillin)	1492		0.0	0.9	0.38	0.41		-	-	-	-
29	Acetylvanillone	1498		-	-	-	-		0.0	0.2	0.10	0.08
30	4-Methyl-2,6-bis(1,1-dimethylethyl)-phenol	1522		0.7	1.3	0.98	0.32		-	-	-	-
31	Dibenzyl	1526		0.0	0.4	0.18	0.17		-	-	-	-
32	Methyl 4-hydroxy-3-methoxy benzoate	1530		0.9	2.9	1.65	0.87		0.1	0.5	0.28	0.17
33	Methyl 4-methoxybenzoate	1546		2.0	3.3	2.50	0.53		0.3	1.0	0.50	0.36
34	Methyl 2,5-dihydroxybenzoate	1551		0.0	1.0	0.45	0.42		0.0	0.3	0.13	0.15
35	4-Hydroxybenzoic acid	1571		0.4	3.7	2.08	1.88		-	-	-	-
36	1,4-Dimethylindanyl acetate	1661		1.8	2.7	2.15	0.55		0.7	1.6	1.03	0.39
37	3,4-Dimethoxybenzoic acid	1672		-	-	-	-		0.3	1.0	0.78	0.32
38	3-Ethyl-2-(but-3-enyl)-cyclohex-2-en-1-one^*^	1705		0.0	1.1	0.70	0.50		0.0	1.5	0.68	0.67
39	Methyl syringate	1788		4.5	6.8	6.03	1.05		3.8	4.2	4.00	0.16
40	Vomofoliol	1820		10.7	13.8	12.15	1.28		21.6	24.2	22.95	1.06
41	Hexadecan-1-ol	1892		0.0	0.5	0.30	0.22		0.5	1.4	0.95	0.47
42	Dibuthyl phtalate	1975		0.3	0.4	0.35	0.06		0.0	1.0	0.68	0.46
43	Hexadecanoic acid	1988		1.0	1.6	1.38	0.26		0.3	1.1	0.78	0.34
44	Pinocembrin	2039		0.0	0.5	0.20	0.25		-	-	-	-
45	(*Z*)-Octadec-9-en-1-ol	2073		1.5	5.5	3.25	1.66		1.0	2.7	1.35	0.90
46	Heneicosane	2100		0.0	2.9	1.78	1.25		0.0	0.2	0.10	0.10
47	(*Z*)-Octadec-9-enoic acid	2181		1.2	3.2	2.15	0.82		0.5	0.8	0.68	0.15
48	Octadecanoic acid	2209		0.0	0.6	0.25	0.30		0.0	0.2	0.10	0.10
49	Tricosane	2300		0.5	1.8	0.83	0.66		0.1	0.2	0.15	0.06

RI = retention indices on HP-5MS column; A = USE with mixture of pentane and diethyl ether (1:2 v/v); B = USE with dichloromethane; - = not detected; ^*^ - tentatively identified; ^**^ - correct isomer not identified.

Since fresh honey samples were analyzed, only a few furan derivatives were found (Table 2): pantoic lactone (-; 0.0-0.1%), 4,5-dimethyl-2-formylfuran (0.1%–0.5%; 0.1%–0.6%) and methyl 2-furoate (0.1%–0.3%; 0.2%–0.7%). Higher aliphatic acids and alcohols were also represented, including hexadecan-1-ol (0.0%–0.5%; 0.5%–1.4%), hexadecanoic acid (1.0%–1.6%; 0.3%–1.1%), (*Z*)-octadec-9-en-1-ol (1.5%–5.5%; 1.0%–2.7%), (*Z*)-octadec-9-enoic acid (1.2%–3.2%; 0.5%–0.8%), octadecanoic acid (0.0%–0.6%; 0.0%–0.2%). Two high-molecular saturated hydrocarbons were found: heneicosane (0.0%–2.9%; 0.0%–0.2%) and tricosane (0.5%–1.8%; 0.1%–0.2%).

### 2.3. Potential Chemical Biomarkers of *P. mahaleb* Honey

**Figure 1:**
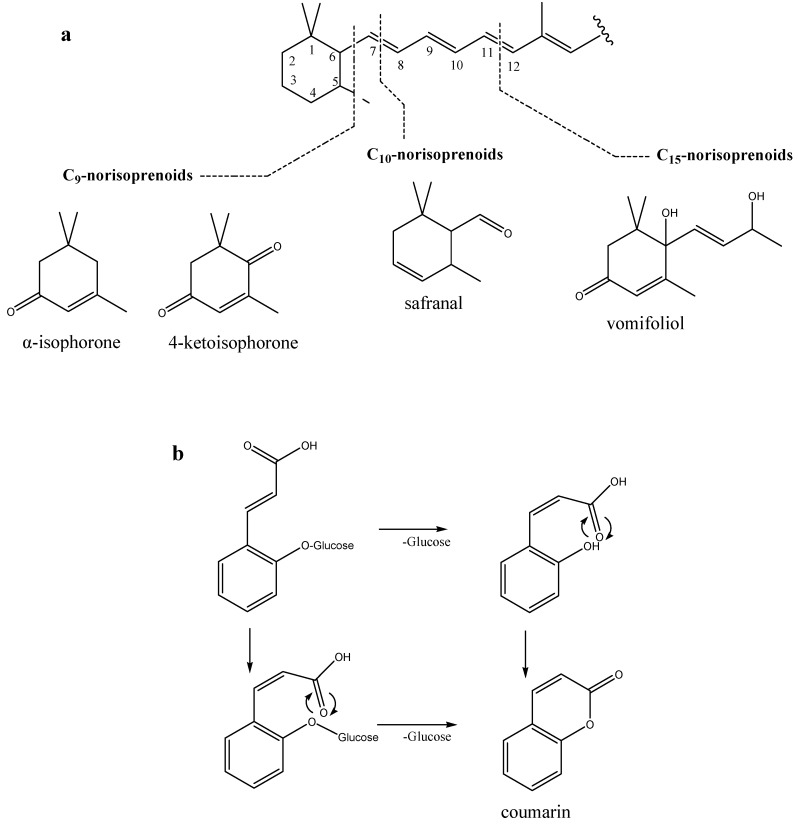
**(a)** Structures of identified norisoprenoids derived from carotenoids. **(b)** Biosynthesis of coumarin from the corresponding glucoside.

One of the objectives of volatiles screening is to identify specific or nonspecific chemical biomarkers (particularly phytochemicals) of the honey’s botanical origin. Compounds with high percentages are of particular interest, even if not specific and/or exclusive for the plant source since their different concentrations could be used for distinguishing among the honey types. Figure 1. presents selected structures of the most prominent volatile organic compounds used for characterization of *P. mahaleb* honey. 

Screening of *P. mahaleb* honey volatiles revealed the presence of the C_15_-norisoprenoid vomifoliol with high percentages in both extracts (Table 1) followed by minor percentages of C_9_- and C_13_-norisoprenoids, mainly in the headspace. Previous studies showed that the types and amounts of norisoprenoids, a class of carotenoid-derived compounds with 3,5,5-trimethylcyclohex-2-enic structures, are related to the honey’s botanical origin, and have been proposed as chemical markers of honey floral source. Some norisoprenoids, such as α-isophorone and dehydrovomifoliol, have been suggested by many authors as markers for heather honeys (*Calluna vulgaris* L.) [11]. However, high percentages of vomifoliol can be considered characteristic for *P. mahaleb* honey, but not exclusively, since we also found vomifoliol to be abundant in *Mentha* spp. honey volatiles [17]. In addition, in our previous research [6], norisoprenoids were isolated by hydrodistillation with minor percentages in the flowers, leaves and bark of *P. mahaleb*, particularly C_13_-norisoprenoids (*E*)-β-damascenone, (*E*)-α-ionone and (*E*)-β-ionone, as well as the C_9_-norisoprenoid α-isophorone. 

Coumarin (*2H*-chromen-2-one), found in the extracts and headspace (Table 1 and Table 2), is another individual compound that can be highlighted in the scope of *P. mahaleb* honey discrimination from other honey types. Coumarin is not common among honey volatile compounds, but it is found in the honeys of *Lavandula angustifolia* Mill., *L. angustifolia × latifolia* and *L. hybrida* Reverchon II, where it served, among others, as a freshness indicator [18,19]. A possible biosynthetic pathway of coumarin generation is lactonisation of *cis*-coumarinic acid liberated by the hydrolysis from the corresponding β-glucoside [19]. In addition, coumarin is a pleasantly fragrant chemical compound found in different parts of *P. mahaleb* plant [6]: bark (34.1%), flowers (2.3%), leaves (5.1%) and wood (4.5%). 

Although linalool derived isomers of lilac aldehyde and/or lilac alcohol were found in the headspace of *P. mahaleb* honey, they are dominant volatiles from nodding thistle honey, citrus honey and *Paliurus spina-christi* Mill. honey [20,21,22] and therefore cannot be considered specific.

## 3. Experimental

### 3.1. Honey Samples

Six *Prunus mahaleb* L. samples were obtained from professional beekeepers (north Croatia) and investigated directly (no mechanical treatment or heat was used). The combs were placed in the area of wild growing *P. mahaleb* L. Melissopalynological analysis was performed by the methods recommended by the International Commission for Bee Botany [23]. Microscopical examination was carried out on a Hund h 500 (Wetzlar, Germany) light microscope attached to a digital camera (Motic m 1000) and coupled to an image analysis system (Motic Images Plus software) for morphometry of pollen grains. All the samples were stored in hermetically closed glass bottles at 4 °C.

### 3.2. Headspace Solid-Phase Microextraction (HS-SPME)

The isolation of headspace volatiles was performed using a manual SPME fiber with the layer of polydimethylsiloxane/divinylbenzene (PDMS/DVB) obtained from Supelco Co (Bellefonte, PA, USA). The fiber was conditioned prior to use according to the manufacturer instructions. For HS-SPME extraction, honey/saturated water solution (5 mL, 1:1 v/v; saturated with NaCl) was placed in a 15 mL glass vial and hermetically sealed with PTFE/silicone septa. The vial was maintained in a water bath at 60 °C during equilibration (15 min) and extraction (45 min) and was partially submerged so that the liquid phase of the sample was below the water level. All the experiments were performed under constant stirring (1,000 rpm) with a magnetic stirrer. After sampling, the SPME fiber was withdrawn into the needle, removed from the vial, and inserted into the injector (250 °C) of the GC and GC-MS for 6 min where the extracted volatiles were thermally desorbed directly to the GC column.

### 3.3. Ultrasonic Solvent Extraction (USE)

Ultrasound-assisted solvent extraction (USE) was performed in an ultrasound cleaning bath (Elmasonic Typ S 30 H, Germany) in indirect sonication mode (sweep mode), at a frequency of 37 kHz at 25 ± 3 °C. Forty grams of each honey sample was dissolved in distilled water (22 mL) in a 100-mL flask. Magnesium sulfate (1.5 g) was added and each sample was extensively vortexed. A mixture of pentane-diethyl ether (1:2, v/v) and dicholoromethane were separately used as the extraction solvent for each honey sample. Sonication was maintained for 30 min. After sonication, the organic layer was separated by centrifugation and filtered over anhydrous MgSO4. The aqueous layer was returned to the flask and another batch of the same extraction solvent (20 mL) was added and extracted by ultrasound for 30 min. The organic layer was separated in the same way as the previous one and filtered over anhydrous MgSO4, and the aqueous layer was sonicated a third time for 30 min with another batch (20 mL) of the extraction solvent. Joined organic extracts were concentrated to 0.2 mL by distillation with a Vigreaux column, and 1 μL was used for GC and GC-MS analyses.

### 3.4. Gas Chromatography and Mass Spectrometry (GC, GC-MS)

Gas chromatography analyses were performed on a Agilent Technologies (Palo Alto, CA, USA) gas chromatograph model 7890A equipped with flame ionization detector, mass selective detector, model 5975C and an Agilent J&W HP-5MS ((5%-phenyl)-methylpolysiloxane capillary GC column (30 m, 0.25 mm i.d., coating thickness 0.25 μm). Chromatographic conditions were as follows: helium was carrier gas at 1 mL·min^−1^, injector temperature was 250 °C, and FID detector temperature was 300 °C. HP-5MS column temperature was programmed at 70 °C isothermal for 2 min, and then increased to 200 °C at a rate of 3 °C·min^−1 ^and held isothermal for 18 min. The injected volume was 1 μL and the split ratio was 1:50. MS conditions were: ionization voltage 70 eV; ion source temperature 230 °C; mass scan range: 30–300 mass units. The analyses were carried out in duplicate.

### 3.5. Data Analysis and Data Evaluation

The individual peaks were identified by comparison of their retention indices (relative to C9-C25 *n-*alkanes for HP-5MS) to those of authentic samples and literature [24], as well as by comparing their mass spectra with the Wiley 275 MS library (Wiley, New York, USA) and NIST02 (Gaithersburg, MD, USA) mass spectral database. The percentage composition of the samples was computed from the GC peak areas using the normalization method (without correction factors). The component percentages (Table 1 and Table 2) were calculated as mean values from duplicate GC and GC-MS analyses.

## 4. Conclusions

Screening of *P. mahaleb* honey was focused on the analyses of natural organic volatiles and their usefulness for honey sourcing. Identified compounds included terpenes, norisoprenoids, benzene derivatives, followed by minor percentages of aliphatic compounds and furan derivatives. High vomifoliol percentages in both extracts and coumarin in the headspace and extracts were considered characteristic for *P. mahaleb* honey and highlighted as potential nonspecific biomarkers useful for honey type discrimination. In addition, comparison with *P. mahaleb* flowers, leaves, bark and wood volatiles from our previous research revealed common compounds among the norisoprenoid and benzene derivatives. 
